# Expression of *FAD* and *SAD* Genes in Developing Seeds of Flax Varieties under Different Growth Conditions

**DOI:** 10.3390/plants13070956

**Published:** 2024-03-26

**Authors:** Elena N. Pushkova, Liubov V. Povkhova, Ekaterina M. Dvorianinova, Roman O. Novakovskiy, Tatiana A. Rozhmina, Aleksey A. Gryzunov, Elizaveta A. Sigova, Daiana A. Zhernova, Elena V. Borkhert, Anastasia A. Turba, Arthur G. Yablokov, Nadezhda L. Bolsheva, Alexey A. Dmitriev, Nataliya V. Melnikova

**Affiliations:** 1 Engelhardt Institute of Molecular Biology, Russian Academy of Sciences, 119991 Moscow, Russia; pushkova18@gmail.com (E.N.P.); povhova.lv@phystech.edu (L.V.P.); dvorianinova.em@phystech.edu (E.M.D.); 0legovich46@mail.ru (R.O.N.); tatyana_rozhmina@mail.ru (T.A.R.); sigova.ea@phystech.edu (E.A.S.); zhernova.d@ya.ru (D.A.Z.); sashai@inbox.ru (E.V.B.); anastas.turba@gmail.com (A.A.T.); melarsoprol@mail.ru (A.G.Y.); nlbolsheva@mail.ru (N.L.B.); 2 Moscow Institute of Physics and Technology, 141701 Moscow, Russia; 3 Federal Research Center for Bast Fiber Crops, 172002 Torzhok, Russia; 4 All-Russian Scientific Research Institute of Refrigeration Industry—Branch of V.M. Gorbatov Federal Research Center for Food Systems of Russian Academy of Sciences, 127422 Moscow, Russia; grizu-nov@rambler.ru; 5 Faculty of Biology, Lomonosov Moscow State University, 119234 Moscow, Russia

**Keywords:** *Linum usitatissimum*, flax, fatty acids, *SAD*, *FAD*, gene expression, transcriptome sequencing

## Abstract

Flax seed is one of the richest plant sources of linolenic acid (LIN) and also contains unsaturated linoleic acid (LIO) and oleic acid (OLE). Stearoyl-ACP desaturases (SADs) and fatty acid desaturases (FADs) play key roles in the synthesis of flax fatty acids (FAs). However, there is no holistic view of which genes from the *SAD* and *FAD* families and at which developmental stages have the highest expression levels in flax seeds, as well as the influence of genotype and growth conditions on the expression profiles of these genes. We sequenced flax seed transcriptomes at 3, 7, 14, 21, and 28 days after flowering (DAF) for ten flax varieties with different oil FA compositions grown under three temperature/watering conditions. The expression levels of 25 genes of the *SAD*, *FAD2*, and *FAD3* families were evaluated. *FAD3b*, *FAD3a*, *FAD2b-2*, *SAD3-1*, *SAD2-1*, *SAD2-2*, *SAD3-2*, *FAD2a-1*, and *FAD2a-2* had the highest expression levels, which changed significantly during seed development. These genes probably play a key role in FA synthesis in flax seeds. High temperature and insufficient watering shifted the maximum expression levels of *FAD* and *SAD* genes to earlier developmental stages, while the opposite trend was observed for low temperature and excessive watering. Differences in the *FAD* and *SAD* expression profiles under different growth conditions may affect the FA composition of linseed oil. Stop codons in the *FAD3a* gene, resulting in a reduced LIN content, decreased the level of *FAD3a* transcript. The obtained results provide new insights into the synthesis of linseed oil.

## 1. Introduction

Flax (*Linum usitatissimum* L.) seed is one of the richest plant sources of omega-3 fatty acids, which are essential for humans and prevent the onset and progression of many diseases [1,2,3,4]. Flax varieties differ significantly in the fatty acid (FA) composition of the oil, especially in the content of linolenic acid (omega-3) [5,6,7]. Traditional flax varieties are high in linolenic acid (LIN, 50–60%) and are used in the pharmaceutical and paint industries [6,8]. Varieties with low (about 5%) and medium (30–40%) LIN contents have also been developed and are promising for use in the food industry due to the greater resistance of oil to rancidity [6,8,9]. Linoleic acid (LIO, omega-6) and oleic acid (OLE, omega-9) are also important unsaturated fatty acids in flax seeds. There is an inverse relationship between LIO and LIN levels: varieties with high LIN contents have low LIO contents, and varieties with low LIN contents have high LIO contents [7,10]. The differences in the OLE content between varieties are less pronounced and can be about 2-fold [7,10]. Varieties with higher OLE contents are promising for the production of oxidation-stable edible oil, chemical feedstock, and biodiesel [8,9].

Desaturases are known to play a key role in the synthesis of flax fatty acids by introducing double bonds into the hydrocarbon chain. Stearoyl-ACP desaturases (SAD) catalyze the conversion of stearic acid (STE) to oleic acid, fatty acid desaturases 2 (FAD2) catalyze the conversion of oleic acid to linoleic acid, and fatty acid desaturases 3 (FAD3) catalyze the conversion of linoleic acid to linolenic acid [11,12,13,14,15,16,17]. In flax, 25 genes of the *SAD* and *FAD* families were identified: *SAD2-1*, *SAD2-2*, *SAD3-1*, *SAD3-2*, *FAD2a-1*, *FAD2a-2*, *FAD2b-1*, *FAD2b-2*, *FAD2c-1*, *FAD2c-2*, *FAD2d-1*, *FAD2d-2*, *FAD2e-1*, *FAD2e-2*, *FAD2f-1*, *FAD2f-2*, *FAD2g-1*, *FAD2g-2*, *FAD2h*, *FAD3a*, *FAD3b*, *FAD3c-1*, *FAD3c-2*, *FAD3d-1*, and *FAD3d-2* [18,19]. However, it is probable that not all of these genes play an important role in linseed oil synthesis; some are practically not expressed in seeds but have rather high expression levels in other organs of flax plants [18,19].

Several studies investigated changes in the expression levels of the *FAD* and *SAD* genes in flax seeds during development. As a result, the stages at which these genes were most active were identified: 20 days after flowering (DAF) for *FAD3* family genes [17]; 16, 22, and 30 DAF for genes of *SAD*, *FAD2*, and *FAD3* families with variations among genotypes [20]; 20 and 24 DAF for genes of *SAD*, *FAD2*, and *FAD3* families [21]; and 15 and 20 DAF for genes of the *FAD2* family [8]. In general, it is clear that the expression levels of *FAD* and *SAD* genes were the highest 2–4 weeks after flowering. However, data from different studies are somewhat divergent, which can be explained by differences in genotypes and growth conditions. Most importantly, in all of the above-mentioned studies, the transcript levels were evaluated for only some of the 25 *FAD* and *SAD* genes known in flax. Therefore, it is difficult to make generalizations about the expression of *FAD* and *SAD* genes in flax seeds during development.

The effect of genotype on the transcript levels of *FAD* and *SAD* genes in flax seeds was also evaluated. Missense and nonsense mutations in the *FAD3a* and *FAD3b* genes are known to result in a reduced LIN content in oil [7,16,17,22,23,24,25,26]. The association of decreased *FAD3a* and *FAD3b* transcript levels with nonsense mutations in these genes was reported [16,17,20]. However, no change in the *FAD3b* transcript level was observed in genotypes with the missense mutation in this gene, leading to a decrease in the LIN content [17]. Furthermore, no correlation was found between the expression levels of *SAD1*, *SAD2*, *FAD2a*, *FAD2b*, *FAD3a*, and *FAD3b* and the FA composition of linseed oil in genotypes with the same desaturase isoforms but different FA compositions [21].

In addition to the genotype, it is known that the environment has an influence on the linseed oil content and its FA composition; in particular, an elevated temperature with deficient moisture led to an increase in the OLE content, whereas a decreased temperature with excess moisture had the opposite effect, leading to an increase in the LIO and LIN contents [10,27,28,29,30]. However, it is currently unclear how growth conditions affect the expression profiles of specific *FAD* and *SAD* genes in flax seeds during development, and whether different genotypes respond similarly to growing in different environments at the level of *FAD* and *SAD* gene expression. We chose contrasting temperature and watering conditions, which led to multidirectional changes in the FA composition of linseed oil, to evaluate the maximum expression variations of *FAD* and *SAD* genes in flax seeds.

Since genes of the *FAD* and *SAD* families play a key role in determining the most important trait of linseed, namely the oil FA composition, it is necessary to understand, as much as possible, which of these genes can contribute the most to the synthesis of linseed oil fatty acids and how genotype and conditions alter their expression profiles. Finding answers to these questions was the focus of our work.

## 2. Results

### 2.1. Results of Transcriptome Sequencing of Flax Seeds

The sequencing of 266 flax seed transcriptome libraries was performed. Ten flax varieties, which differed in oil FA composition and the presence of mutations in the *FAD3* genes, leading to a reduced LIN content, were used in this work (Table 1): high-LIN AGT 427, Atalante, Entre-Rios, Norlin, and Pechersky kryazh; mid-LIN Raciol and AGT 422; and low-LIN AGT 981, AGT 1535, and Lola. The seeds were collected at 3, 7, 14, 21, and 28 days after flowering (DAF) from plants grown under conditions of 16 °C with overwatering (hereafter referred to as 16 °C), 20 °C with optimal watering (hereafter referred to as 20 °C), and 24 °C with underwatering (hereafter referred to as 24 °C). No data were obtained for the variety AGT 422 at 21 and 28 DAF at 24 °C and for the variety Lola at 28 DAF at 24 °C due to the negative impacts of elevated temperature and insufficient watering on these genotypes. Sequencing was performed in two biological replicates for the samples collected at 3, 7, 14, and 21 DAF and in one biological replicate for the samples collected at 28 DAF (because of the difficulty of obtaining high-quality RNA from seeds at 28 DAF). On average, 2 million paired-end reads were generated for each transcriptome library.

To visualize the relatedness of the studied samples, they were clustered based on the expression of all identified transcripts. The samples were grouped mainly by developmental stage (i.e., DAF) and growth conditions (16 °C, 20 °C, and 24 °C) rather than by their belonging to a specific genotype (Figure S1).

### 2.2. Expression of FAD and SAD Genes in Flax Seeds

The expression of *FAD* and *SAD* family genes, known to play an important role in linseed FA synthesis [14,16,18,19,23,26,31,32], was evaluated. From 25 flax genes of the *SAD* and *FAD* families (*SAD2-1*, *SAD2-2*, *SAD3-1*, *SAD3-2*, *FAD2a-1*, *FAD2a-2*, *FAD2b-1*, *FAD2b-2*, *FAD2c-1*, *FAD2c-2*, *FAD2d-1*, *FAD2d-2*, *FAD2e-1*, *FAD2e-2*, *FAD2f-1*, *FAD2f-2*, *FAD2g-1*, *FAD2g-2*, *FAD2h*, *FAD3a*, *FAD3b*, *FAD3c-1*, *FAD3c-2*, *FAD3d-1*, and *FAD3d-2*) [18,19,26], the highest expression levels in the seeds were found for *FAD3b*, *FAD3a*, *FAD2b-2*, *SAD3-1*, *SAD2-1*, *SAD2-2*, *SAD3-2*, *FAD2a-1*, and *FAD2a-2* (Supplementary Table S1). It can be assumed that these genes play the most important roles in linseed oil synthesis among the *FAD* and *SAD* genes. For these genes, a more detailed analysis of the expression profiles in different flax varieties grown at 16 °C, 20 °C, and 24 °C was performed.

### 2.3. Expression Profiles of FAD3a and FAD3b Genes during Flax Seed Development under Different Temperature and Watering Conditions

**Figure 1:**
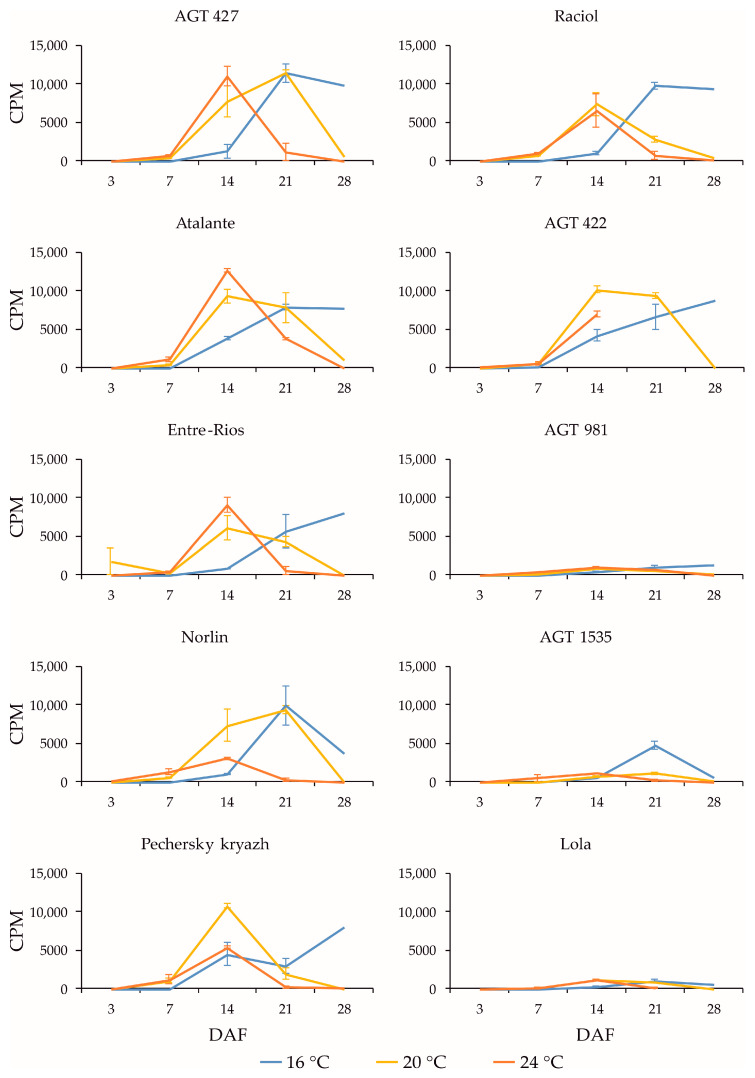
Expression profiles (3, 7, 14, 21, and 28 DAF) of *FAD3a* gene in high-LIN (AGT 427, Atalante, Entre-Rios, Norlin, and Pechersky kryazh), mid-LIN (Raciol and AGT 422), and low-LIN (AGT 981, AGT 1535, and Lola) flax varieties grown at 16 °C and overwatered (16 °C), at 20 °C and optimally watered (20 °C), and at 24 °C and underwatered (24 °C). Data are missing for 21 and 28 DAF at 24 °C for AGT 422 and 28 DAF at 24 °C for Lola. Error bars represent values obtained for two biological replicates.

**Figure 2:**
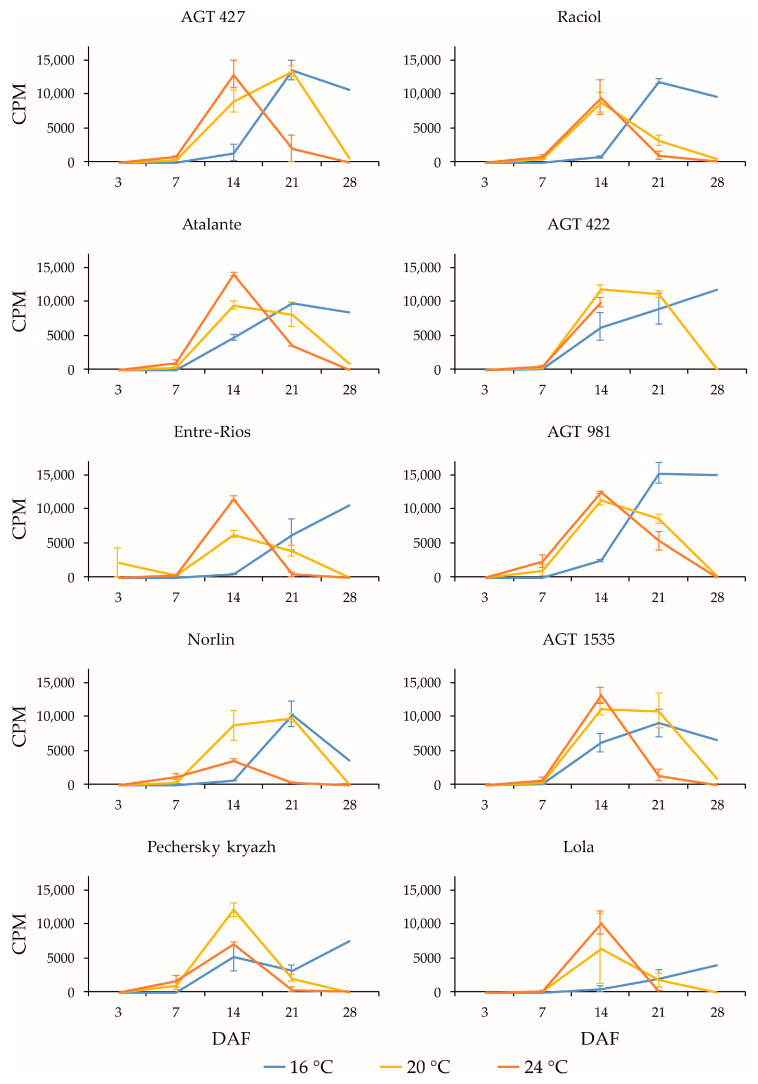
Expression profiles (3, 7, 14, 21, and 28 DAF) of *FAD3b* gene in high-LIN (AGT 427, Atalante, Entre-Rios, Norlin, and Pechersky kryazh), mid-LIN (Raciol and AGT 422), and low-LIN (AGT 981, AGT 1535, and Lola) flax varieties grown at 16 °C and overwatered (16 °C), at 20 °C and optimally watered (20 °C), and at 24 °C and underwatered (24 °C). Data are missing for 21 and 28 DAF at 24 °C for AGT 422 and 28 DAF at 24 °C for Lola. Error bars represent values obtained for two biological replicates.

*FAD3* genes are known to play a key role in the conversion of LIO to LIN in flax seeds [16,17]. Among the *FAD3* genes, the expression of *FAD3a* and *FAD3b* was the highest in the studied samples; it was ten times higher than the expression of *FAD3c-1*, *FAD3c-2*, *FAD3d-1*, and *FAD3d-2.* In different flax varieties, the expression of *FAD3a* and *FAD3b* generally changed in a similar manner during seed development under the same growth conditions (Figure 1 and Figure 2, respectively). The expression was minimal at 3 DAF. The maximum expression levels of the *FAD3a* and *FAD3b* genes at 16 °C were observed at 21 or 28 DAF in most varieties. At 20 °C, the maximum expression levels were reached at 14 or 21 DAF, after which a decrease was observed. At 24 °C, the maximum expression levels of the *FAD3a* and *FAD3b* genes were reached at 14 DAF, after which a decrease was observed. It is noteworthy that the maximum expression levels of the *FAD3a* and *FAD3b* genes were similar at 16 °C, 20 °C, and 24 °C. Thus, an increased temperature resulted in a shift in the maximum level of gene expression to earlier stages of seed development, while a decreased temperature resulted in a shift to later stages of seed development.

The expression profiles of the *FAD3a* and *FAD3b* genes were very similar for the same variety under the same conditions (16 °C, 20 °C, and 24 °C). However, in the varieties AGT 981, AGT 1535, and Lola, the level of *FAD3a* transcript was drastically lower than in the other varieties, while the level of *FAD3b* transcript was similar to the other varieties. The varieties AGT 981, AGT 1535, and Lola are known to have mutations in the *FAD3a* gene, resulting in stop codons and a reduced LIN content [7]. Thus, the *FAD3a* transcript level was reduced in varieties with nonsense mutations in this gene. The situation was different for the *FAD3b* gene. The varieties AGT 422, AGT 981, AGT 1535, and Raciol, which carry the *FAD3b* mutation, leading to a histidine-to-tyrosine substitution in exon 2 and a reduction in the LIN content [7], had approximately the same expression level of this gene as the varieties without the mutation. It is worth noting that the expression level of *FAD3b* was slightly higher than that of *FAD3a* in all of the varieties studied.

### 2.4. Expression Profiles of FAD2b-2, FAD2a-1, and FAD2a-2 Genes during Flax Seed Development under Different Temperature and Watering Conditions

**Figure 3:**
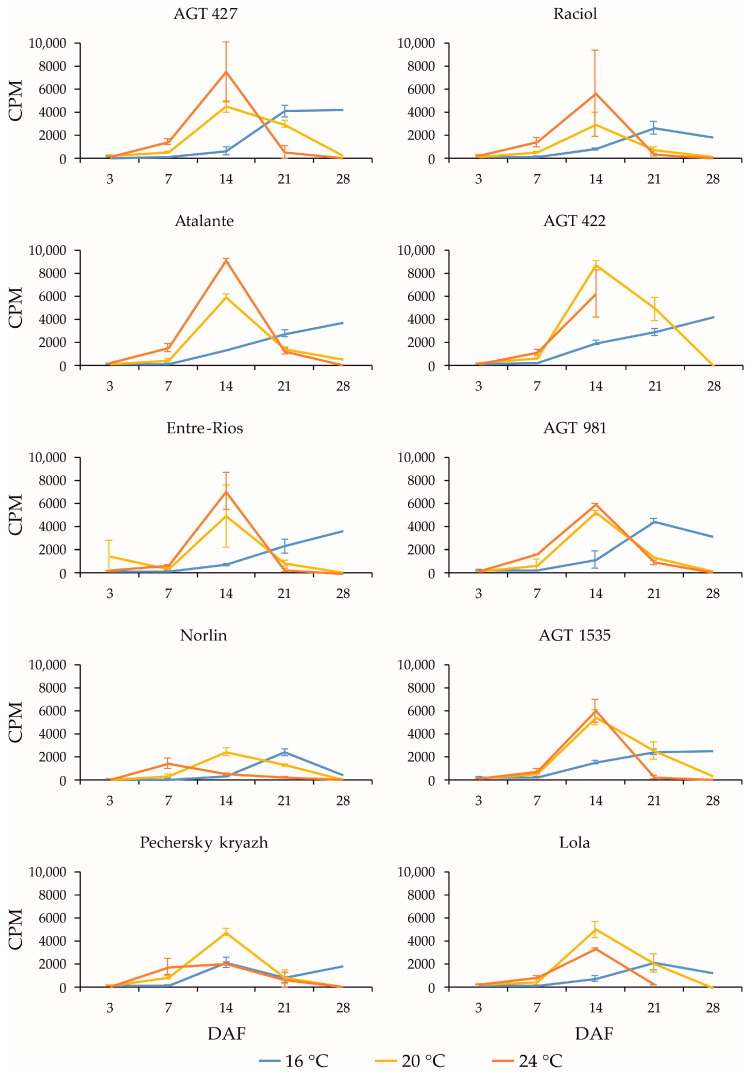
Expression profiles (3, 7, 14, 21, and 28 DAF) of *FAD2b-2* gene in high-LIN (AGT 427, Atalante, Entre-Rios, Norlin, and Pechersky kryazh), mid-LIN (Raciol and AGT 422), and low-LIN (AGT 981, AGT 1535, and Lola) flax varieties grown at 16 °C and overwatered (16 °C), at 20 °C and optimally watered (20 °C), and at 24 °C and underwatered (24 °C). Data are missing for 21 and 28 DAF at 24 °C for AGT 422 and 28 DAF at 24 °C for Lola. Error bars represent values obtained for two biological replicates.

*FAD2* genes play an important role in the synthesis of linoleic acid from oleic acid [14,15]. Among *FAD2* genes, the highest expression levels in flax seeds were found for *FAD2b-2*, *FAD2a-1*, and *FAD2a-2* (Supplementary Table S1). The expression level of *FAD2b-2* was several times higher than those of *FAD2a-1* and *FAD2a-2* in all varieties studied. The expression level of *FAD2a-1* was slightly higher than that of *FAD2a-2*. It should be noted that the *FAD2a-1* and *FAD2a-2* genes have very similar sequences, which differ by only seven SNPs, which could introduce some bias in the estimation of their individual expression patterns [19]. For *FAD2b-2* (Figure 3), *FAD2a-1* (Supplementary Figure S2), and *FAD2a-2* (Supplementary Figure S3), as for *FAD3a* and *FAD3b*, cultivation at 16 °C resulted in altered expression profiles compared to those at 20 °C and 24 °C and a shift in the maximum expression levels to later stages of seed development (21 or 28 DAF). The expression profiles of *FAD2a-1* and *FAD2a-2* were very similar for the same variety under the same conditions, but slightly different from that of *FAD2b-2*.

All varieties showed drastic changes in the expression levels of *FAD2b-2*, *FAD2a-1*, and *FAD2a-2* during seed development. At 3 DAF, *FAD2b-2*, *FAD2a-1*, and *FAD2a-2* were barely expressed, but at 14 DAF (for 20 °C and 24 °C) or later (for 16 °C), a dramatic increase in expression levels was observed. At 28 DAF, there was a strong decrease in the expression levels of the genes for all varieties at 20 °C and 24 °C, and there were differences in the expression changes (increase, decrease, or retention compared to 21 DAF) among varieties at 16 °C. A decrease in the maximum expression level of *FAD2b-2* at 16 °C compared to 20 °C and/or 24 °C could also be observed for some varieties. Among the 10 examined flax varieties, Pechersky kryazh had the highest OLE content (31.8%), which was 1.5–2.0 times higher than that in other varieties. In this variety, the expression level of *FAD2b-2* was slightly lower than that in the majority of studied varieties. The expression level of *FAD2b-2* was also slightly lower in Norlin, which had an increased OLE content (22.2%), and Lola, which had a decreased OLE content (12.9%). For *FAD2a-1* and *FAD2a-2*, the differences in expression levels between Pechersky kryazh and other varieties were not very significant. At the same time, the expression profiles of *FAD2b-2*, *FAD2a-1*, and *FAD2a-2* in Pechersky kryazh were slightly different from those of other varieties, and the maximum expression levels were shifted to earlier stages of seed development (this was clearly visible for *FAD2a-1* and *FAD2a-2* at 20 °C and for *FAD2b-2* at 24 °C). Thus, the high OLE content in the variety Pechersky kryazh is likely not related to the maximum *FAD2* expression levels. However, the shift in the maximum expression levels of *FAD2* genes to earlier stages of seed development could play a role in increasing the OLE content in linseed oil.

### 2.5. Expression Levels of SAD Genes during Flax Seed Development under Different Temperature and Watering Conditions

**Figure 4:**
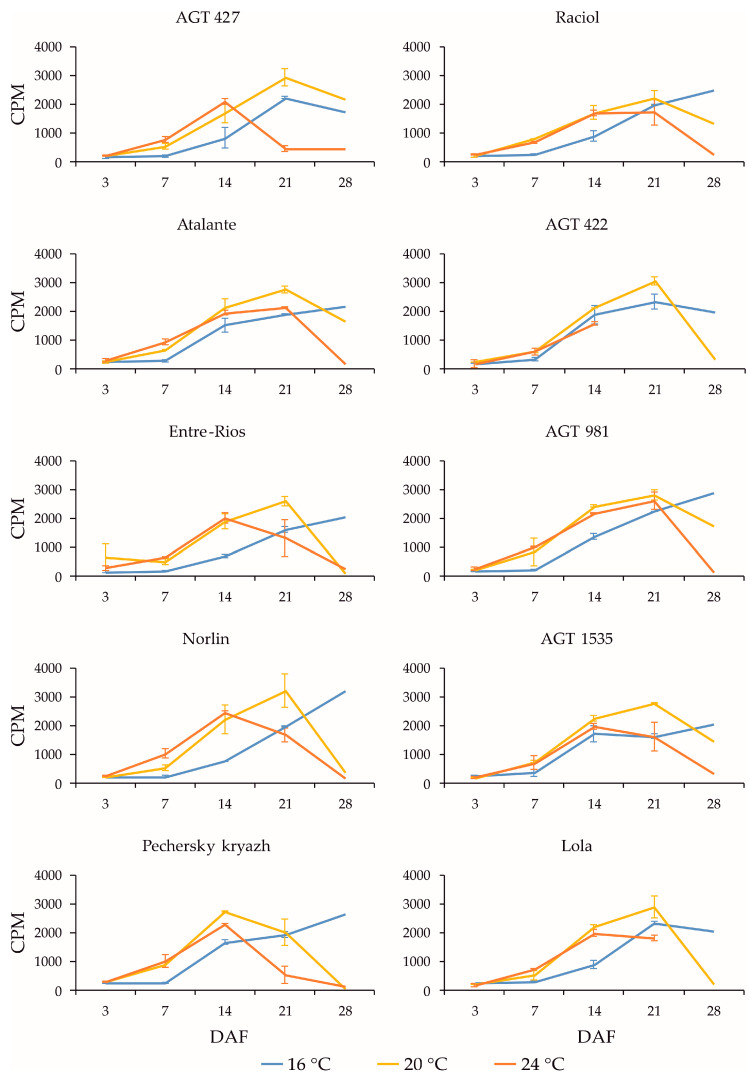
Expression profiles (3, 7, 14, 21, and 28 DAF) of *SAD2-1* gene in high-LIN (AGT 427, Atalante, Entre-Rios, Norlin, and Pechersky kryazh), mid-LIN (Raciol and AGT 422), and low-LIN (AGT 981, AGT 1535, and Lola) flax varieties grown at 16 °C and overwatered (16 °C), at 20 °C and optimally watered (20 °C), and at 24 °C and underwatered (24 °C). Data are missing for 21 and 28 DAF at 24 °C for AGT 422 and 28 DAF at 24 °C for Lola. Error bars represent values obtained for two biological replicates.

**Figure 5:**
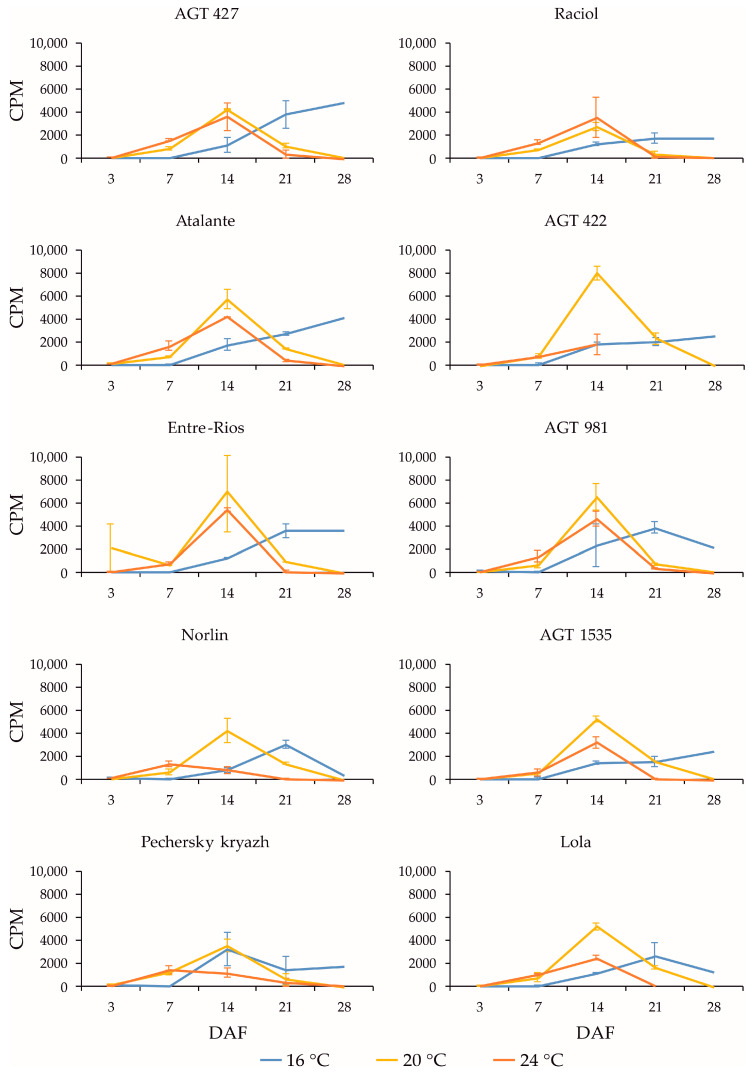
Expression profiles (3, 7, 14, 21, and 28 DAF) of *SAD3-1* gene in high-LIN (AGT 427, Atalante, Entre-Rios, Norlin, and Pechersky kryazh), mid-LIN (Raciol and AGT 422), and low-LIN (AGT 981, AGT 1535, and Lola) flax varieties grown at 16 °C and overwatered (16 °C), at 20 °C and optimally watered (20 °C), and at 24 °C and underwatered (24 °C). Data are missing for 21 and 28 DAF at 24 °C for AGT 422 and 28 DAF at 24 °C for Lola. Error bars represent values obtained for two biological replicates.

*SAD* genes are involved in the conversion of stearic acid to oleic acid in flax seeds [11,12,13]. In flax, all four identified *SAD* genes (*SAD2-1*, *SAD2-2*, *SAD3-1*, and *SAD3-2*) were rather highly expressed （Supplementary Figure S1), with the highest level being observed for *SAD3-1*. The expression profiles of *SAD2-1* (Figure 4) and *SAD2-2* (Supplementary Figure S4) for the same variety under the same conditions were very similar. The same was true for *SAD3-1* (Figure 5) and *SAD3-2* (Supplementary Figure S5). Some differences in expression profiles were observed between the *SAD2-1/SAD2-2* and *SAD3-1/SAD3-2* pairs for the same samples, although the overall trend of expression changes was similar. During seed development, an increase in the expression levels of *SAD* genes was initially observed for all studied conditions. Then, at 20 °C and 24 °C, a decrease in expression levels was observed at 21 or 28 DAF, while at 16 °C, different varieties showed an increase, decrease, or retention of expression levels at 21 and 28 DAF. At the same time, at 20 °C and 24 °C, *SAD3-1* and *SAD3-2* reached maximum expression levels at earlier stages of seed development (14 DAF for most varieties) compared to *SAD2-1* and *SAD2-2* (21 DAF at 20 °C and 14 or 21 DAF at 24 °C for most varieties).

It can be noted that, for *SAD2-1/SAD2-2*, some expression was observed even at 3 DAF, in contrast to the *SAD3-1/SAD3-2* and *FAD* genes, for which expression was practically absent at this stage. It is also interesting that the expression changes during seed development (most notably at 20 °C) were more similar between *SAD3-1/SAD3-2* and *FAD2b-2* (reaching maximum levels at earlier stages) and between *SAD2-1/SAD2-2* and *FAD2a-1/FAD2a-2*, *FAD3a*, and *FAD3b* (reaching maximum levels at later stages). In Pechersky kryazh with a high OLE content in the oil, characteristics similar to those described above for the *FAD2* genes were observed for the *SAD* genes. Thus, the expression levels of *SAD* genes in the high-OLE variety Pechersky kryazh were close to those in the low-OLE variety Lola. However, in Pechersky kryazh, the *SAD* expression profiles were slightly different from those of other varieties, and the maximum expression levels were shifted to earlier stages of seed development (this was particularly noticeable for *SAD2-1* and *SAD2-2* at 20 °C and for *SAD3-1* and *SAD3-2* at 24 °C). Thus, the high OLE content in the variety Pechersky kryazh could be related not to its differences from other varieties in the maximum expression levels of *SAD* and *FAD2* genes, but to the shift in the maximum expression levels of these genes to earlier stages of seed development.

## 3. Discussion

It is known that the key role in the synthesis of unsaturated fatty acids of linseed oil is played by genes of the *SAD*, *FAD2*, and *FAD3* families [11,12,13,14,15,16,17]. We analyzed the expression of these genes during seed development in a representative set of 10 flax varieties with different oil FA compositions grown under three different temperature and watering conditions. In contrast to most previous works evaluating *SAD* and *FAD* gene expression in flax [8,16,17,18,20,21], we obtained individual expression data for all 25 known *SAD* and *FAD* genes rather than the expression levels for only a few genes from each family or common expression patterns for pairs of homologous genes. Due to the large number of works devoted to conducting transcriptome analyses of different flax organs and tissues [33,34,35,36,37,38,39,40,41,42,43,44], we were previously able to determine which of the 25 *SAD* and *FAD* genes are expressed at high levels in flax seeds and to estimate the expression levels of these genes in other organs and tissues [19]. However, in that work, because of the lack of transcriptomic data, we were unable to assess the dynamics of expression of these genes during seed development; thus, this was carried out in the present work for a representative set of flax varieties grown under different conditions affecting the FA composition of linseed oil.

The highest expression levels were detected for *FAD3b*, *FAD3a*, *FAD2b-2*, *SAD3-1*, *SAD2-1*, *SAD2-2*, *SAD3-2*, *FAD2a-1*, and *FAD2a-2.* In addition, the expression levels of these genes changed tens and hundreds of times during seed development. In the early stages of development, the expression of these genes was minimal, and then a drastic increase was observed, but the dynamics of rise depended significantly on the growth conditions. The strongest increase was observed at 20 °C and 24 °C, with a maximum reached at 14 or 21 DAF in most varieties, followed by a drastic decrease. At 24 °C, compared to 20 °C, a shift in the maximum expression levels to earlier developmental stages was observed for the majority of the highly expressed *FAD* and *SAD* genes. At 16 °C, the maximum expression levels of the analyzed genes were observed at later stages of seed development (mainly at 21 and 28 DAF), and the differences between varieties were quite pronounced. It is likely that, among the *FAD* and *SAD* families, *FAD3b*, *FAD3a*, *FAD2b-2*, *SAD3-1*, *SAD2-1*, *SAD2-2*, *SAD3-2*, *FAD2a-1*, and *FAD2a-2* genes with high maximum expression levels and drastic expression changes during seed development play a key role in the synthesis of the FA of linseed oil.

It is also possible to hypothesize how variations in *FAD* and *SAD* expression are reflected in the composition of linseed oil when plants are grown under different temperature and moisture conditions. Wet and cold summers are known to increase the LIN + LIO content in linseed oil, whereas hot and dry summers are known to increase the OLE content [10]. In our study, the decrease in *FAD2a-1* and *FAD2a-2* expression at elevated temperature (24 °C) occurred at earlier stages of seed development compared to 20 °C and, especially, 16 °C. This trend was less clear for *FAD2b-2*, but it was also present in some genotypes. Under conditions of elevated temperature and insufficient watering, the *FAD2* genes probably do not have sufficient time to desaturate oleic acid to linoleic acid to the same extent as at reduced temperature, resulting in increased OLE and decreased LIO+LIN contents. *FAD* genes are known to be involved in the responses to a variety of stresses, including high and low temperatures [45]. The effects of increased and/or decreased temperatures on the expression of *FAD* genes were reported for banana [46], maize [47], *Gossypium* [48], cucumber [49], and some genotypes of soybean [50]. The effect of temperature on the production of LIN and LIO in olive was also shown [51]. For flax, we observed that temperature affects the expression profiles of *FAD* and *SAD* genes, which could be reflected in the oil FA composition.

We did not find a correlation between the expression levels of *FAD2* or *SAD* genes and the OLE content. Thus, among the genotypes we studied, there was the high-OLE variety Pechersky kryazh, and expression levels of *FAD2* and *SAD* genes in it were close to those of the low-OLE variety Lola. At the same time, a shift in the maximum expression levels of *FAD* and *SAD* genes to earlier stages of seed development was observed in Pechersky kryazh compared to the other varieties studied. This peculiarity could lead to a higher content of OLE in the seeds of this variety. The strongest expression of *FAD* and *SAD* genes in this variety may occur in hotter summer periods than in other varieties, which could further enhance the effects of an increasing OLE content and a decreasing LIO content. We grew flax plants under controlled conditions at constant temperatures, so differences in the flowering time did not bias the evaluation of the expression levels. However, when plants are grown under field conditions, the contribution of differences in flowering time between genotypes could also be reflected in the oil FA composition, as seed maturation may occur under different temperature/watering conditions.

Among the *SAD* genes, the highest expression level was found for *SAD3-1*. The expression level of its homolog, *SAD3-2*, was, on average, two times lower. The expression levels of the homologous genes *SAD2-1* and *SAD2-2* were quite high, not more than 30% lower than that of *SAD3-1*. The expression level of *SAD2-1* was slightly higher than that of *SAD2-2*. It can be assumed that the *SAD3-1* gene plays the major role in the conversion of STE to OLE in flax seeds, but *SAD2-1*, *SAD2-2*, and *SAD3-2* also contribute significantly to this process.

Among the *FAD2* genes, *FAD2b-2* had the highest expression level in most samples. However, the expression level of its homolog, *FAD2b-1*, was tens of times lower. For the homologous genes *FAD2a-1* and *FAD2a-2*, the expression levels were quite similar, with a slightly higher level of *FAD2a-1*. *FAD2b-2* is probably the key gene in the desaturation of oleic acid to linoleic acid in flax seeds, but *FAD2a-1* and *FAD2a-2* also contribute to this process. The role of *FAD2b-1* in linoleic acid synthesis in linseed oil is probably insignificant, which is also characteristic of the other *FAD2* genes studied, namely *FAD2c-1*, *FAD2c-2*, *FAD2d-1*, *FAD2d-2*, *FAD2e-1*, *FAD2e-2*, *FAD2f-1*, *FAD2f-2*, *FAD2g-1*, *FAD2g-2*, and *FAD2h*.

Among the *FAD3* genes, *FAD3a* and *FAD3b* were characterized by the highest expression levels. The expression levels of these genes were similar, but in the varieties AGT 981, AGT 1535, and Lola with nonsense mutations in the *FAD3a* gene, leading to a reduced LIN content, the level of the *FAD3a* transcript was ten times lower than the level of the *FAD3b* transcript. The low level of the *FAD3a* transcript in the flax genotype with a mutation in this gene, leading to a stop codon, was previously reported and explained by nonsense-mediated mRNA decay [17]. No decrease in the *FAD3b* transcript level was detected in varieties with a missense mutation in *FAD3b*, leading to a reduction in the LIN content. The fact that a missense mutation in the *FAD3b* gene does not lead to a change in its expression compared to other varieties was also reported previously [17]. It was also shown that several *FAD3a* and *FAD3b* gene isoforms carrying mutations encode non-functional enzymes that are unable to convert LIO to LIN [26]. The contribution of *FAD3a* and *FAD3b* to the formation of LIN from LIO is probably close, but the expression level of *FAD3b* was slightly higher than that of *FAD3a*, so the role of *FAD3b* in the synthesis of linolenic acid might be slightly higher. This assumption is supported by the higher LIN content in flax varieties with inactivating mutations in the *FAD3a* gene compared to varieties with inactivating mutations in the *FAD3b* gene [7].

We observed similar expression profiles for *SAD3-1*, *SAD3-2*, and *FAD2b-2.* The expression profiles of *SAD2-1*, *SAD2-2*, *FAD2a-1*, *FAD2a-2*, *FAD3a*, and *FAD3b* were also similar. For the first group of genes, maximum expression levels were reached at earlier stages of seed development compared to the second group. There may be common regulatory mechanisms for the genes in each group, and these groups of genes may contribute somewhat differently to the synthesis of linseed oil at different stages of seed development.

Thus, by analyzing more than two hundred cDNA libraries of flax seeds at five stages of development for a representative set of ten flax varieties grown under three different environmental conditions, we were able to identify the key genes and stages of the FA synthesis of linseed oil and evaluate the influence of environmental conditions and genotype on the expression of these genes. *FAD* and *SAD* genes with the highest expression in flax seeds were identified, and these genes probably play important roles in the synthesis of OLE, LIO, and LIN in flax seeds. *SAD3-1*, *SAD2-1*, *SAD2-2*, and *SAD3-2* contribute to the synthesis of OLE from STE. Among the fifteen *FAD2* genes, the *FAD2b-2* gene is probably the key gene in the desaturation of OLE to LIO, but *FAD2a-1* and *FAD2a-2* also contribute to this process. Among the six *FAD3* genes, *FAD3a* and *FAD3b* play major roles in the conversion of LIO to LIN. The obtained data, in addition to their basic significance, are important for the selection of *FAD* and *SAD* genes for editing to obtain flax varieties with modified FA compositions to meet the requirements of different economic sectors. For the *FAD3a* gene, a correlation was found between the transcript level and the LIN content, which was associated with nonsense mutations in this gene. This feature could be used in the marker-assisted selection of flax to develop varieties with reduced linolenic acid contents for the food industry. The analyzed *FAD* and *SAD* genes showed dramatic changes in the expression levels during seed development, and the expression profiles of these genes were quite similar during seed development under the same growth conditions. At the same time, the growth conditions contributed significantly to the expression profiles: under elevated temperature and insufficient watering, maximum expression levels were reached at earlier stages, and an earlier decrease in expression during seed development was observed, while reduced temperature and excessive watering shifted the maximum expression levels to later stages of seed development. Thus, 14 DAF was the key for FA synthesis in flax seeds at 24 °C with underwatering, and 14 and 21 DAF were the keys at 20 °C with optimal watering, while 21 and 28 DAF turned out to be the most important at 16 °C with overwatering. These expression changes could be reflected in the FA composition of linseed oil. In addition, genotype-dependent features of *FAD* and *SAD* expression profiles were found, which were more pronounced at 16 °C with overwatering. Moreover, the shift in expression profiles to earlier stages of seed development in the variety Pechersky kryazh compared to the other varieties could result in a high OLE content in its linseed oil under certain conditions. Thus, our work is important for understanding the contribution of genotype and environment to the expression of *FAD* and *SAD* genes, which can be used to develop recommendations for optimal conditions of linseed cultivation to obtain oil with a specific FA composition.

## 4. Materials and Methods

### 4.1. Flax Varieties

We used plants of 10 flax varieties: AGT 422, AGT 427, AGT 981, AGT 1535, Atalante, Entre-Rios, Lola, Norlin, Pechersky kryazh (namely, l. 1-2 from k-2889 according to the catalog of the Federal Research Center for Bast Fiber Crops, selected from Pechersky kryazh, originating from N.I. Vavilov All-Russian Institute of Plant Genetic Resources (VIR)), and Raciol. AGT 981, AGT 1535, and Lola had low LIN contents; Raciol and AGT 422 had medium LIN contents; and AGT 427, Entre-Rios, Norlin, Atalante, and Pechersky kryazh had high LIN contents (Table 1). Low and medium LIN levels were associated with mutations in the *FAD3a* and *FAD3b* genes [7]; the data are shown in Table 1.

### 4.2. Growth Conditions

Flax plants were grown in 15 L pots with soil pre-treated with fungicide. pH was maintained at about 5.5, which is the optimum level for flax. Thirty-two seeds were sown per pot, and three pots were planted for each variety. All plants were maintained under the same conditions for one month after planting. The plants were then transferred to three climate chambers with different temperature regimes for growth. In the first chamber, the temperature was maintained at 16 °C and watering was carried out every day. In the second chamber, the temperature was 20 °C and watering was carried out every two days. In the third chamber, the temperature was 24 °C and watering was carried out every three days.

### 4.3. Collection of Plant Material and RNA Isolation

The flower was marked with the date of the day it opened (day of flowering) and the data were recorded in a table. Seeds were collected at 3, 7, 14, 21, and 28 days after flowering (DAF). For each variety, at least 10 samples were collected for each temperature/watering condition and each developmental stage. Seeds collected from individual capsules were placed in tubes and immediately frozen in liquid nitrogen. Plant material was placed in a low-temperature freezer at −70 °C.

For RNA isolation, seeds were first ground to a fine powder using disposable homogenization pestles (Helicon, Moscow, Russia) inserted into a DeWalt DCD701D2 cordless drill (DeWalt, Towson, MD, USA) at a speed of 1200–1500 rpm in 1.5 mL tubes in liquid nitrogen without thawing the sample. RNA isolation was performed according to the protocol of Wang et al. [52] with some modifications. Briefly, 1 mL CTAB lysis buffer (2% CTAB (neoFroxx GmbH, Einhausen, Germany), 2% PVP K30 (PanReac AppliChem, Darmstadt, Germany), 100 mM Tris HCl pH 8.0 (Thermo Fisher Scientific, Waltham, MA, USA), 25 mM EDTA (Thermo Fisher Scientific), 2 M NaCl (Scharlab, Barcelona, Spain), and 2% β-mercaptoethanol (Bio-Rad, Hercules, CA, USA)) pre-warmed to 65 °C were added to the homogenized sample. The homogenate was incubated in a TDB-120 thermostat (Biosan, Riga, Latvia) at 65 °C for 30 min with mixing every 10 min. Then, five identical samples (same variety, growth conditions, and developmental stage) were mixed as follows: 350 μL of homogenate was taken from each sample using a 1000 mL wide-bore pipette tip and added to a 2 mL tube. Next, 500 μL of homogenate was taken from the pool (the remainder was frozen in liquid nitrogen), and 500 μL of CTAB lysis buffer pre-warmed to 65 °C was added. In the next step, an equal volume of cold chloroform (Acros Organics, Geel, Belgium) was added to the homogenate, vortexed for 30 s, and then centrifuged for 20 min at 10,000× *g* and 4 °C in a 5418R microcentrifuge (Eppendorf, Hamburg, Germany). The aqueous phase was transferred to clean tubes, and then an equal volume of cold chloroform was added, vortexed for 30 s, and centrifuged for 10 min at 10,000× *g* and 4 °C. The aqueous phase was transferred to clean tubes, and 1/2 volume of 96% ethanol was added and mixed until homogeneous. Total RNA was then purified using the CleanRNA Standard Kit (Evrogen, Moscow, Russia) according to the manufacturer’s protocol with a DNAase I treatment step from the RNase-Free DNase Set (Qiagen, Chatsworth, CA, USA). RNA quality and concentration were evaluated using a gel electrophoresis, 2100 Bioanalyzer (Agilent Technologies, Santa Clara, CA, USA), and Qubit 4 fluorometer (Thermo Fisher Scientific). Only non-degraded RNA samples with a concentration of at least 20 ng/μL were used for further work.

### 4.4. Preparation and Sequencing of cDNA Libraries

cDNA libraries for sequencing of flax seed transcriptomes were prepared using the QIAseq Stranded mRNA Select Kit (Qiagen) according to the manufacturer’s protocol. RNA from seeds of 10 flax varieties (AGT 422, AGT 427, AGT 981, AGT 1535, Atalante, Entre-Rios, Lola, Norlin, Pechersky kryazh, and Raciol) collected at 3, 7, 14, 21, and 28 DAF from plants grown under three different temperature/watering conditions were used. For 3, 7, 14, and 21 DAF, cDNA libraries were prepared in 2 replicates, and for 28 DAF, cDNA libraries were prepared in 1 replicate (because of the difficulty of obtaining RNA of high quality and sufficient quantity for seeds of late developmental stages). The quality of the obtained cDNA libraries (agreement of the length of the obtained libraries with the expected ones and the absence of adapter dimers) was evaluated on a 2100 Bioanalyzer (Agilent Technologies), and the concentration was evaluated on a Qubit 4 fluorometer (Thermo Fisher Scientific).

The resulting cDNA libraries (266 libraries in total, as the libraries for the variety AGT 422 at 21 and 28 DAF at 24 °C and the variety Lola at 28 DAF at 24 °C were excluded from the analysis because of their low quality) were mixed equimolarly and sequenced on a NextSeq 2000 instrument (Illumina, San Diego, CA, USA) using the NextSeq 2000 P3 Reagents (100 Cycles) kit (Illumina) in 51 + 51 nucleotide format.

### 4.5. Expression Analysis

Raw reads were processed using Trimmomatic 0.38 [53]. The data were checked for the presence of adapter sequences, trimmed for quality (TRAILING:24 and SLIDINGWINDOW:4:14), and filtered for length (MINLEN:40). The PPline tool was then used for expression analysis [54]. Transcriptomic reads were aligned to the variety Atlant genome (GCA_014858635.1 in NCBI Genome) [55], and counts per million (CPM) values were calculated for 25 genes: *SAD2-1* (Atlant transcript H1233_031351), *SAD2-2* (H1233_038408), *SAD3-1* (H1233_054424), *SAD3-2* (H1233_039970), *FAD2a-1* (H1233_058938), *FAD2a-2* (H1233_061927), *FAD2b-1* (H1233_075799), *FAD2b-2* (H1233_078572), *FAD2c-1* (H1233_075801), *FAD2c-2* (H1233_078571), *FAD2d-1* (H1233_041596), *FAD2d-2* (H1233_078570), *FAD2e-1* (H1233_075803), *FAD2e-2* (H1233_078569), *FAD2f-1* (H1233_075804), *FAD2f-2* (H1233_078567), *FAD2g-1* (H1233_075805), *FAD2g-2* (H1233_078566), *FAD2h* (H1233_078565), *FAD3a* (H1233_027729), *FAD3b* (H1233_038272), *FAD3c-1* (H1233_054813), *FAD3c-2* (H1233_039845), *FAD3d-1* (H1233_041596), and *FAD3d-2* (H1233_041890).

## Figures and Tables

**Table 1:** Characteristics of flax varieties used in this work.

Variety	OLE, %	LIO, %	LIN, %	*FAD3a* Mutation G to A (Lu7:16092348)	*FAD3b* Mutation C to T (Lu12:1035655)	*FAD3a* Mutation C to T (Lu7:16090340)
AGT 427	13.1	13.4	64.3	−	−	−
Atalante	16.4	14.7	58.2	−	−	−
Entre-Rios	20.1	16.5	52.5	−	−	−
Norlin	22.2	14.5	54.3	−	−	−
Pechersky kryazh	31.8	10.2	52.0	−	−	−
Raciol	15.5	39.2	35.0	−	+	−
AGT 422	18.3	35.5	32.6	−	+	−
AGT 981	18.5	67.2	3.2	+	+	−
AGT 1535	18.8	64.0	4.9	+	+	−
Lola	12.9	68.0	9.4	−	−	+

Note: The coordinates of the mutation sites are given according to the *L. usitatissimum* genome assembly GCA_000224295.2/ASM22429v2. Data on the FA composition and mutations are taken from references [7,10].

## Data Availability

The raw sequencing data have been deposited in the NCBI Sequence Read Archive (SRA) under the BioProject accession number PRJNA1039849.

## Supplementary Materials

The following supporting information can be downloaded at https://www.mdpi.com/article/10.3390/plants13070956/s1, Figure S1: Clustering of seed samples collected at five time points (3 (3d), 7 (7d), 14 (14d), 21 (21d), and 28 (28d) days after flowering) for ten flax varieties (AGT 422 (422), AGT 427 (427), AGT 981 (981), AGT 1535 (1535), Atalante (Ata), Entre-Rios (Ent), Lola (Lol), Norlin (Nor), Pechersky kryazh (Pech), and Raciol (Rac)) grown under three different temperature/watering conditions (16 °C and overwatering (16C), 20 °C and optimal watering (20C), 24 °C and underwatering (24C)) based on gene expression profiles. Figure S2: Expression profiles (3, 7, 14, 21, and 28 DAF) of *FAD2a-1* gene in high-LIN (AGT 427, Atalante, Entre-Rios, Norlin, and Pechersky kryazh), mid-LIN (Raciol and AGT 422), and low-LIN (AGT 981, AGT 1535, and Lola) flax varieties grown at 16 °C and overwatered (16 °C), at 20 °C and optimally watered (20 °C), and at 24 °C and underwatered (24 °C). Figure S3: Expression profiles (3, 7, 14, 21, and 28 DAF) of *FAD2a-2* gene in high-LIN (AGT 427, Atalante, Entre-Rios, Norlin, and Pechersky kryazh), mid-LIN (Raciol and AGT 422), and low-LIN (AGT 981, AGT 1535, and Lola) flax varieties grown at 16 °C and overwatered (16 °C), at 20 °C and optimally watered (20 °C), and at 24 °C and underwatered (24 °C). Figure S4: Expression profiles (3, 7, 14, 21, and 28 DAF) of *SAD2-2* gene in high-LIN (AGT 427, Atalante, Entre-Rios, Norlin, and Pechersky kryazh), mid-LIN (Raciol and AGT 422), and low-LIN (AGT 981, AGT 1535, and Lola) flax varieties grown at 16 °C and overwatered (16 °C), at 20 °C and optimally watered (20 °C), and at 24 °C and underwatered (24 °C). Figure S5: Expression profiles (3, 7, 14, 21, and 28 DAF) of *SAD3-2* gene in high-LIN (AGT 427, Atalante, Entre-Rios, Norlin, and Pechersky kryazh), mid-LIN (Raciol and AGT 422), and low-LIN (AGT 981, AGT 1535, and Lola) flax varieties grown at 16 °C and overwatered (16 °C), at 20 °C and optimally watered (20 °C), and at 24 °C and underwatered (24 °C); Table S1: Expression levels of *SAD* (*SAD2-1*, *SAD2-2*, *SAD3-1*, and *SAD3-2*), *FAD2* (*FAD2a-1*, *FAD2a-2*, *FAD2b-1*, *FAD2b-2*, *FAD2c-1*, *FAD2c-2*, *FAD2d-1*, *FAD2d-2*, *FAD2e-1*, *FAD2e-2*, *FAD2f-1*, *FAD2f-2*, and *FAD2g-2*), and *FAD3* (*FAD3a*, *FAD3b*, *FAD3c-1*, *FAD3c-2*, *FAD3d-1*, and *FAD3d-2*) genes in seeds (3, 7, 14, 21, and 28 DAF) of flax plants of 10 varieties (AGT 422 (422), AGT 427 (427), AGT 981 (981), AGT 1535 (1535), Atalante (Ata), Entre-Rios (Ent), Lola (Lol), Norlin (Nor), Pechersky kryazh (Pech), and Raciol (Rac)) grown under different temperature and watering conditions (16 °C, 20 °C, and 24 °C).
