# Supplementary material for: Expression of FAD and SAD Genes in Developing Seeds of Flax Varieties under Different Growth Conditions
Source: Plants (Basel). 2024 Mar 26;13(7):956. doi: 10.3390/plants13070956 (PMC11013676; doi:10.3390/plants13070956)
Supplement: Supplementary file 1 [file plants-13-00956-s001.zip › FigS4_SAD2-2_2024.03.23.pdf]

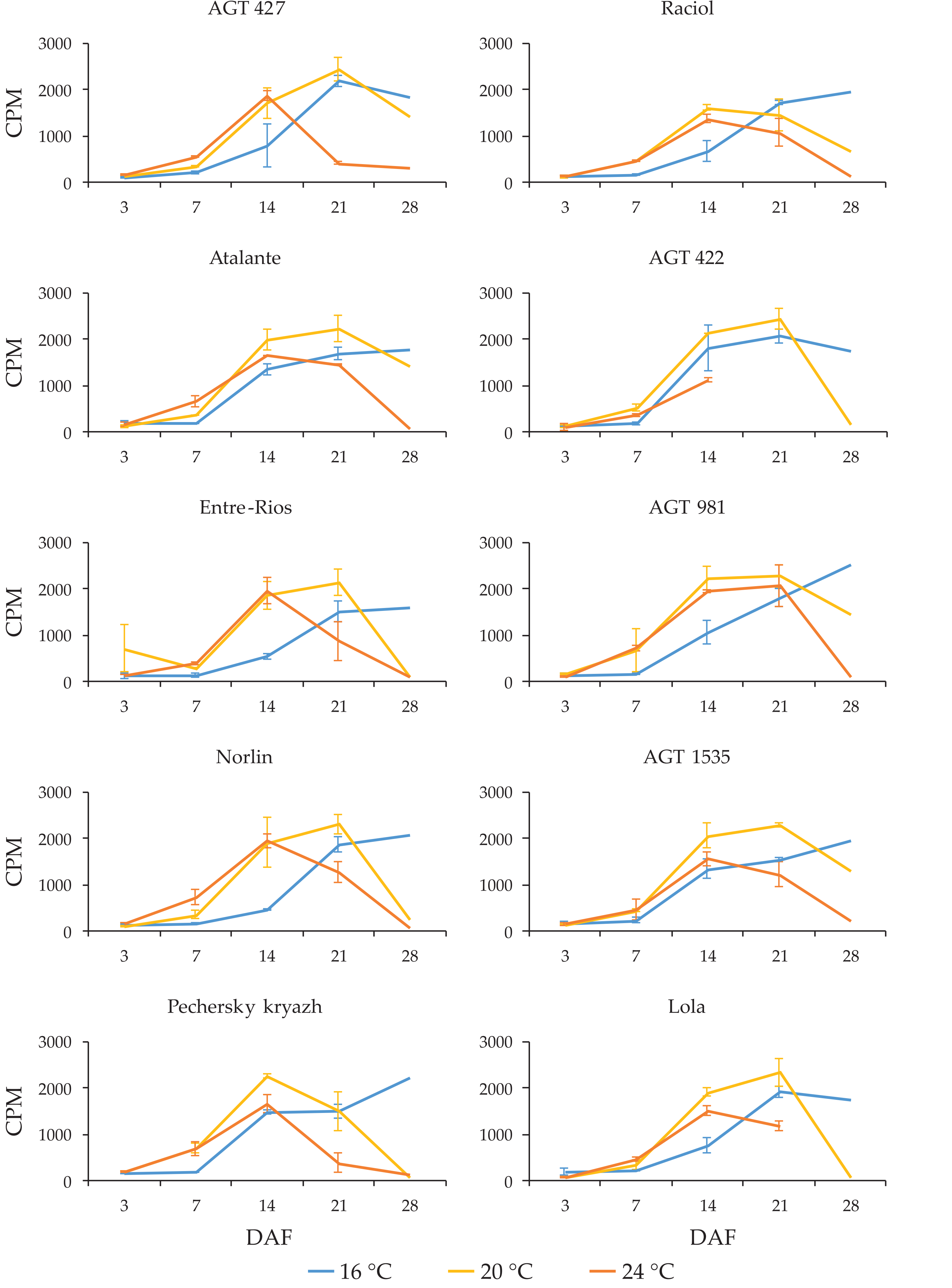

Figure S4. Expression profiles (3, 7, 14, 21, and 28 DAF) of *SAD2-2* gene in high-LIN (AGT 427, Atalante, Entre-Rios, Norlin, and Pechersky kryazh), mid-LIN (Raciol and AGT 422), and low-LIN (AGT 981, AGT 1535, and Lola) flax varieties grown at 16 °C and overwatered (16 °C), at 20 °C and optimally watered (20 °C), and at 24 °C and underwatered (24 °C). Data are missing for 21 and 28 DAF at 24 °C for AGT 422 and 28 DAF at 24 °C for Lola. Error bars represent values obtained for two biological replicates.
