# Supplementary figures and images for: Expression of FAD and SAD Genes in Developing Seeds of Flax Varieties under Different Growth Conditions
Source: Plants (Basel). 2024 Mar 26;13(7):956. doi: 10.3390/plants13070956 (PMC11013676; doi:10.3390/plants13070956)

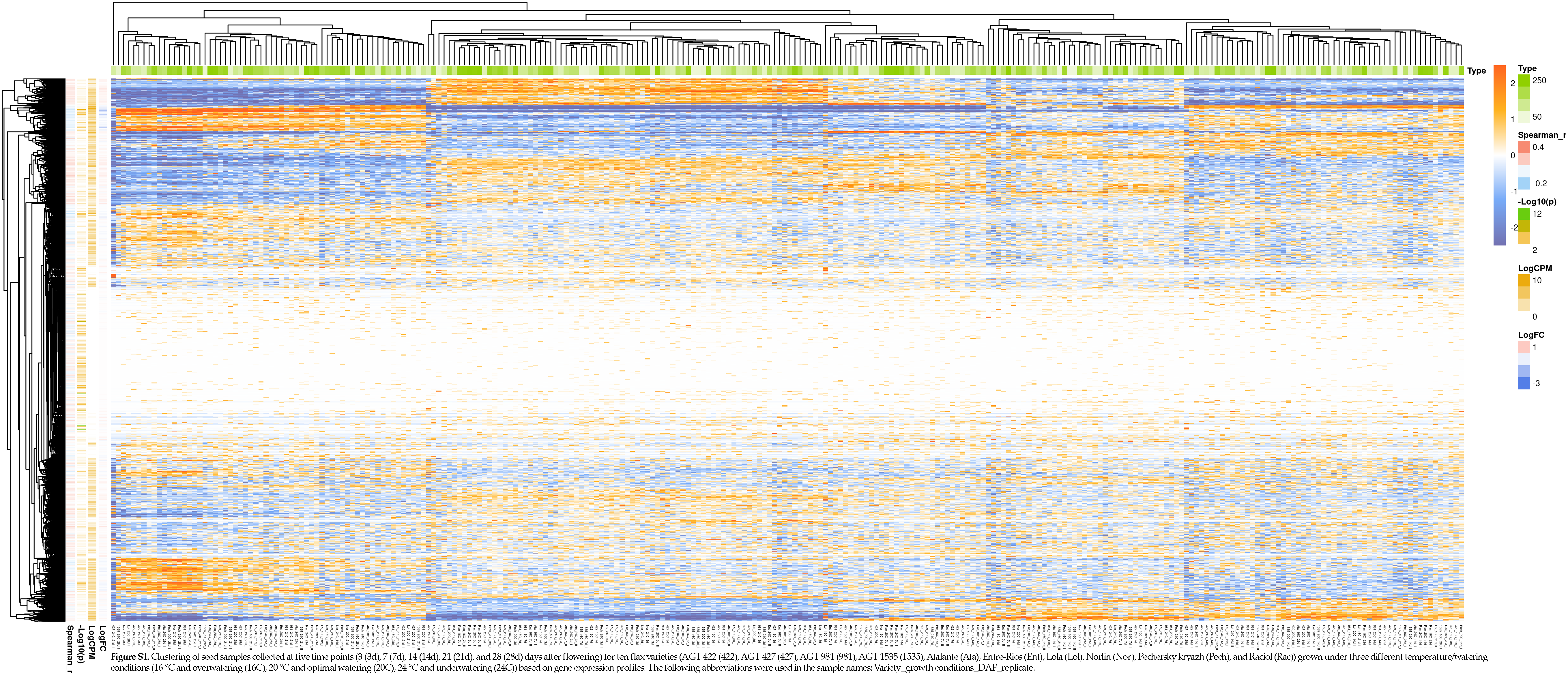

Supplement: Supplementary file 1 [file plants-13-00956-s001.zip › FigS1_Heatmap_expression_2024.03.25.png]
